# Reduction of Obesity and Insulin Resistance through Dual Targeting of VAT and BAT by a Novel Combination of Metabolic Cofactors

**DOI:** 10.3390/ijms232314923

**Published:** 2022-11-29

**Authors:** Sergio Quesada-Vázquez, Anna Antolín, Marina Colom-Pellicer, Gerard Aragonès, Laura Herrero, Josep Maria Del Bas, Antoni Caimari, Xavier Escoté

**Affiliations:** 1 Eurecat, Centre Tecnològic de Catalunya, Unitat de Nutrició i Salut, 43204 Reus, Spain; 2 Nutrigenomics Research Group, Department of Biochemistry and Biotechnology, Universitat Rovira i Virgili, 43007 Tarragona, Spain; 3 Department of Biochemistry and Physiology, School of Pharmacy and Food Sciences, Institut de Biomedicina de la Universitat de Barcelona (IBUB), Universitat de Barcelona, 08028 Barcelona, Spain; 4 Centro de Investigación Biomédica en Red de Fisiopatología de la Obesidad y la Nutrición (CIBEROBN), Instituto de Salud Carlos III, 28029 Madrid, Spain; 5 Eurecat, Centre Tecnològic de Catalunya, Àrea de Biotecnologia, 43204 Reus, Spain

**Keywords:** obesity, adipose tissue, insulin resistance, thermogenesis, metabolic cofactors

## Abstract

Obesity is an epidemic disease worldwide, characterized by excessive fat accumulation associated with several metabolic perturbations, such as metabolic syndrome, insulin resistance, hypertension, and dyslipidemia. To improve this situation, a specific combination of metabolic cofactors (MC) (betaine, N-acetylcysteine, L-carnitine, and nicotinamide riboside) was assessed as a promising treatment in a high-fat diet (HFD) mouse model. Obese animals were distributed into two groups, orally treated with the vehicle (obese + vehicle) or with the combination of metabolic cofactors (obese + MC) for 4 weeks. Body and adipose depots weights; insulin and glucose tolerance tests; indirect calorimetry; and thermography assays were performed at the end of the intervention. Histological analysis of epidydimal white adipose tissue (EWAT) and brown adipose tissue (BAT) was carried out, and the expression of key genes involved in both fat depots was characterized by qPCR. We demonstrated that MC supplementation conferred a moderate reduction of obesity and adiposity, an improvement in serum glucose and lipid metabolic parameters, an important improvement in lipid oxidation, and a decrease in adipocyte hypertrophy. Moreover, MC-treated animals presented increased adipose gene expression in EWAT related to lipolysis and fatty acid oxidation. Furthermore, MC supplementation reduced glucose intolerance and insulin resistance, with an increased expression of the glucose transporter *Glut4*; and decreased fat accumulation in BAT, raising non-shivering thermogenesis. This treatment based on a specific combination of metabolic cofactors mitigates important pathophysiological characteristics of obesity, representing a promising clinical approach to this metabolic disease.

## 1. Introduction

The World Health Organization (WHO) defines obesity as an epidemic disease caused by an imbalance in energy consumption and storage, characterized by an expansion of adipose tissue, which negatively affects health and healthcare systems worldwide [1]. Obesity is a complex disease associated with several comorbidities including metabolic syndrome, insulin resistance (IR), hypertension, and dyslipidemia [2]. It is imperative to find new strategies to fight against this disease. Current therapies focus on increasing physical activity and reducing excess calorie intake that causes energy imbalance, but with low adherence by the general population, or include invasive approaches such as bariatric surgery [3]. Adipose tissue is especially important due to its capacity to secrete several substances that regulate metabolic pathways, such as hormones, cytokines, and adipokines [4]. When adipocytes reach their maximal fat storage capacity and insulin fails to promote the appropriate storage of more fatty acids due to IR, lipolysis is activated in adipocytes, and lipids are released as free fatty acids (FFA) into the circulatory system [5] and are deposited in other peripheral tissues where they may produce lipotoxicity [6,7].

Mardinoglu et al. [8] performed personal model-assisted identification of NAD+ and glutathione (GSH) metabolism to elucidate mechanisms underlying NAFLD, a disease strongly related to obesity, and to discover which substances could be used as treatments due to their participation in the affected metabolic pathways. Prior studies have described how different bioactive co-factors separately related to GSH and NAD^+^ metabolism can modulate metabolic pathways and improve white adipose tissue dysfunction in preclinical models [9,10,11,12,13,14,15]. N-acetylcysteine (NAC) and betaine were selected because both ingredients are GSH precursors [16,17], and nicotinamide riboside (NR), which is a precursor of NAD^+^, and L-carnitine were employed to boost fatty acid oxidation promoting the uptake of FFA to mitochondria, thus accelerating FFA oxidation and protecting against impaired mitochondrial function [18,19,20]. NAC improved glucose and insulin tolerance by reducing insulin levels in HFD-fed mice [10]. After betaine administration, IR was ameliorated [12] and the dysfunctional lipolysis in adipose tissue was restored [20]. L-carnitine demonstrated its boosting function in FFA transport to the mitochondrial matrix [21], whereas L-carnitine deficiency reduces FFA oxidation and increases lipolysis in adipose tissue [14]. Finally, NR is an NAD^+^ donor essential for maintaining a cellular redox state and producing energy for oxidative metabolism [11]. NR in combination with L-carnitine reduced mice’s visceral and subcutaneous WAT depots [21]. Thus, this work evaluated for the first time the beneficial effects on adipose tissue of this novel combination of metabolic cofactors. Taking into consideration the importance of adipose dysfunction in metabolic disorders and in light of previous studies we hypothesized that these metabolic cofactors in combination could activate fatty acid oxidation and FFA uptake in adipocytes by improving IR in an obese animal model. To validate this hypothesis, we analyzed the administration of a combination of MC that included NAC, NR, L-carnitine, and betaine, for 1 month in a diet-induced obese mouse model [22,23], focusing on the main pathological characteristics of adipose tissues.

## 2. Results

### 2.1. MC Supplementation Alleviates Obese Diet-Induced Obesity

**Figure 1:**
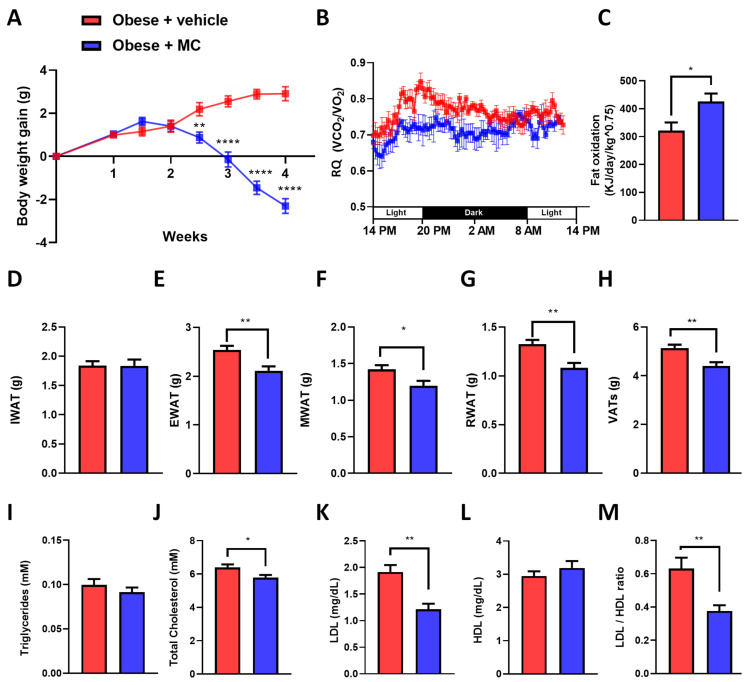
Obese mice supplemented with MC had reduced body weight and smaller visceral WAT depots with an improved biochemical profile. Effects of MC treatment on: (**A**) body weight gain; (**B**) RQ for 24 h; (**C**) fat oxidation. Weights of different adipose tissue depots: (**D**) inguinal (IWAT); (**E**) epididymal (EWAT); (**F**) mesenchymal (MWAT); (**G**) retroperitoneal (RWAT). (**H**) weight of VATs (EWAT + MWAT + RWAT). Serum biochemical parameters of: (**I**) triglycerides; (**J**) total cholesterol; (**K**) LDL-cholesterol; (**L**) HDL-cholesterol; and (**M**) LDL/HDL ratio. Data are mean ± SEM. * *p* < 0.05, ** *p* < 0.01, **** *p* < 0.0001.

Bodyweight gain in the obese + MC group was reduced in comparison with the obese + vehicle group from two and a half weeks of the supplementation (Figure 1A). Similar results were observed in the percentage of body weight gain (Figure S1A). These differences were not the consequences of a reduction in food intake (Figure S1B). Regarding this effect on body weight, indirect calorimetry was performed to analyze lipid oxidation through the analysis of the respiratory quotient (RQ) (Figure 1B,C). As a result, the obese + MC animals showed significantly decreased RQ in comparison with the obese + vehicle, directly related to an increase in fat oxidation. In contrast, no differences were observed regarding energy expenditure (EE) (Figure S2A), oxygen consumption (VO_2_) (Figure S2B), or carbon dioxide production (VCO_2_) (Figure S2C). Due to the increased lipid oxidation observed with MC supplementation, adipose tissue depots were analyzed. However, supplementation of MC did not cause any effect on IWAT (Figure 1D and Figure S1B) although it significantly reduced the visceral WAT depots’ weights (EWAT, RWAT, and MWAT) (Figure 1E–G) and the sum of visceral depots (VAT, Figure 1H) compared with the obese + vehicle group, suggesting that MC could induce a reduction of lipid content in the VAT of obese mice through different processes such as inhibition of adipogenesis or lipogenesis, or an increase of lipolysis or lipid oxidation [24]. Similar results were observed in the percentage of each adipose depot in relation to the total body weight (Figure S1D–G). In addition, biochemical analyses showed no significant differences in serum triglyceride levels (Figure 1I) or HDL levels (Figure 1L). However, significant decreases in circulating total cholesterol (Figure 1J) and LDL-cholesterol levels (Figure 1K) and in the LDL/HDL ratio (Figure 1M) were found in the MC group compared to the obese + vehicle group, suggesting an improvement in the lipid profile of these supplemented mice. 

### 2.2. MC Supplementation Improves Adiposity by Decreasing Adipocyte Hypertrophy in Epididymal Adipose Tissue, with Beneficial Effects on Lipolysis and Fatty Acid Oxidation

**Figure 2:**
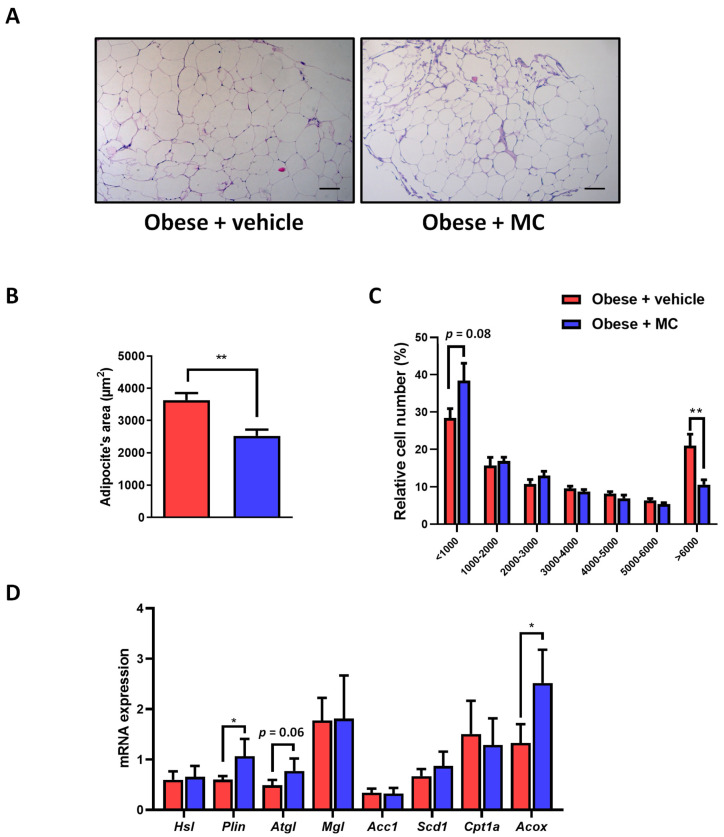
MC supplementation promotes a reduction in the adipocyte size, increasing lipolysis and fatty acid oxidation. Effects of treatments on adipocyte hypertrophy (**A**) Representative micrographs of hematoxylin–eosin stained EWAT sections from obese + vehicle and obese + MC groups (bar = 100 µm); (**B**) adipocyte area; (**C**) adipocyte size distribution; and (**D**) EWAT mRNA expression of genes related to lipolysis (*Hsl*, *Plin*, *Atgl* and *Mgl*), de novo lipogenesis (*Acc1* and *Scd1*), and fatty acid oxidation (*Cpt1a* and *Acox1*). Data are mean ± SEM. * *p* < 0.05, ** *p* < 0.01.

Representative histological images revealed notorious larger adipocytes in the obese + vehicle animals compared with obese + MC animals (Figure 2A). To evaluate these changes, a quantitative analysis of the adipose area was performed (Figure 2B). A reduction in the average adipocyte area was observed in the obese + MC group compared to the obese + vehicle group. the adipocyte size distribution showed a reduction in the percentage of larger adipocytes and a tendency for smaller adipocytes to increase in MC-supplemented mice (Figure 2C).

To determine which metabolic pathways could be involved in improving adipose tissue metabolism after MC supplementation, gene expression was evaluated for genes related to adipose lipolysis, de novo lipogenesis, and fatty acid oxidation (Figure 2D). MC-supplemented mice showed an up-regulation in *Plin1* expression compared to obese + vehicle mice, pointing to a possible effect on lipolytic activity. No significant effects on *Mgl* and *Hsl* expression were observed. In contrast, *Atgl* expression showed a tendency to increase in MC-supplemented animals. On the other hand, MC supplementation did not modify de novo lipogenesis gene expression (*Acc1* and *Scd1*). Interestingly, *Acox1* showed a significant increase in its expression in lipid oxidation after MC supplementation, but no changes were observed in *Cpt1a* expression. 

### 2.3. MC Supplementation Reduced Insulin Resistance Associated with Obesity

**Figure 3:**
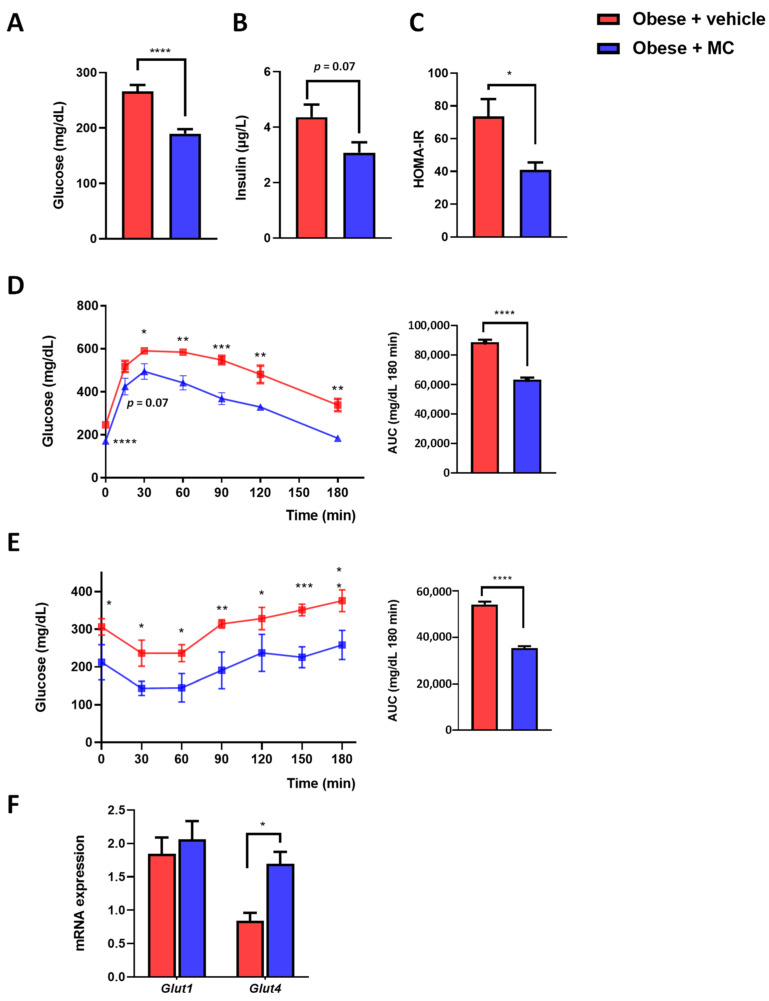
MC supplementation promotes improved glucose and insulin resistance by increasing the expression of *Glut4* in EWAT. (**A**) Fasting glucose; (**B**) fasting insulin; (**C**) HOMA-IR; (**D**) glucose tolerance test (GTT) and area under the curve (AUC); (**E**) insulin tolerance test (ITT) and AUC; (**F**) EWAT mRNA expression of *Glut1* and *Glut4*. Data are mean ± SEM. * *p* < 0.05, ** *p* < 0.01, *** *p* < 0.001, **** *p* < 0.0001.

Fasting glucose (Figure 3A), fasting insulin (Figure 3B), and HOMA-IR (Figure 3C) were determined. The MC group reverted significantly to the increase in circulatory glucose levels observed in the obese + vehicle group, and showed a significant tendency towards reduced circulatory insulin levels. Reductions in glucose and insulin levels were accompanied by a reduction in HOMA-IR [25]. GTT (Figure 3D) and ITT (Figure 3E) were performed to analyze glucose and insulin sensitivity. In both tests, significant differences were observed from basal time until the end of the tests, with an important amelioration in glucose tolerance and IR in the animals supplemented with MC. To validate the amelioration of IR, the EWAT expression was evaluated for key genes in glucose uptake, *Glut1* and *Glut4* (Figure 3F). No significant effects between groups were observed in *Glut1* expression. However, the MC group showed an increased expression of *Glut4* in the EWAT, which could be related to an improvement in IR. 

### 2.4. MC Supplementation Reduces Fat Accumulation in Brown Adipose Tissue by Activation of Lipolysis, Lipid Oxidation and Thermogenesis

**Figure 4:**
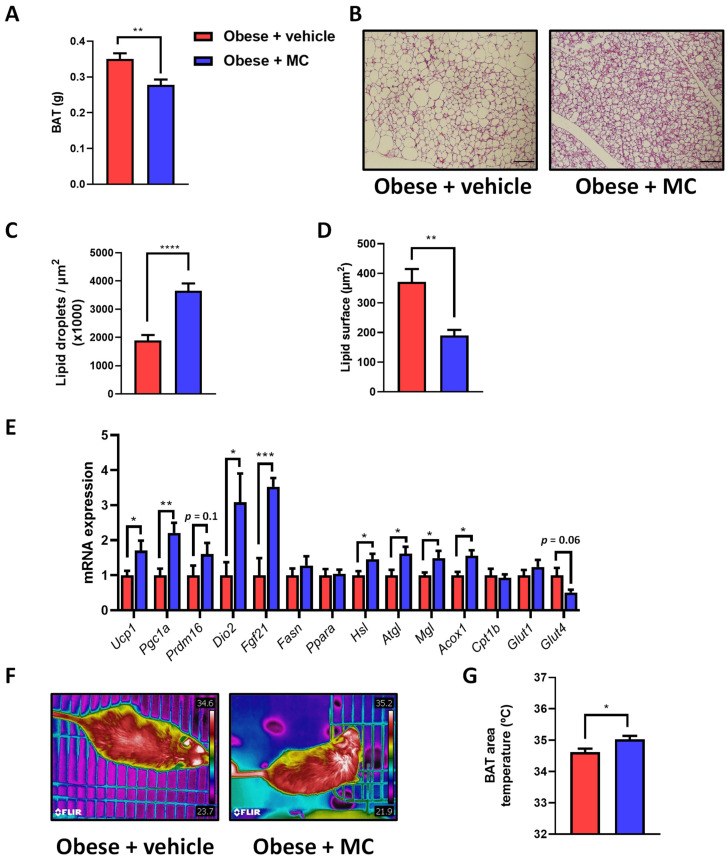
MC supplementation increases BAT thermogenesis. Effects of treatments on: (**A**) brown adipose tissue (BAT) weight; (**B**) representative micrographs of hematoxylin–eosin-stained BAT sections (Bar = 100 µm); (**C**) lipid droplets, and (**D**) and quantification of the lipid droplet surface. (**E**) BAT mRNA expression of genes related to BAT function (*Ucp1*, *Pgc1a*, *Prdm16*, *Dio2*, and *Fgf21*); de novo lipogenesis (*Fasn* and *Ppara*); lipolysis (*Hsl*, *Atgl* and *Mgl*); fatty acid oxidation (*Acox* and *Cpt1b*); and glucose uptake (*Glut1* and *Glut4*). (**F**,**G**) Representative thermographic images of the BAT skin area and quantification. Data are mean ± SEM. * *p* < 0.05, ** *p* < 0.01, *** *p* < 0.001, **** *p* < 0.0001.

BAT activation provides a protective mechanism against excessive body weight and accumulation of fat mass by non-shivering thermogenesis, with important roles in triglyceride clearance, glucose homeostasis, and insulin sensitivity [26]. To better understand the molecular mechanisms associated with the improvement of lipid metabolism, the impact of MC supplementation on BAT was analyzed. BAT weight showed a significant decrease in MC-supplemented mice compared with their counterparts (Figure 4A). Similar results were observed in the percentage of BAT in relation to total body weight (Figure S1H). In addition, histological studies revealed increased fat accumulation in BAT in the obese + vehicle animals compared with obese + MC animals (Figure 4B–D), with a reduction in the number of lipid droplets (Figure 4C) and the lipid surface area (Figure 4D) in the obese + MC animals. 

To determine which metabolic pathways could be involved in the reduction of fat accumulation in BAT after MC supplementation, mRNA expression analysis was carried out for genes related to main BAT functions (Figure 4E). MC-supplemented mice showed an up-regulation in *Ucp1*, *Fgf21*, *Pgc1a* and *Dio2* expression compared with obese + vehicle mice, and a tendency towards increased *Prmd16* levels, pointing to a possible increase in thermogenic capacity. In contrast, no significant effects were observed in the expression of de novo lipogenesis-related genes, *Fasn* and *Ppara*. Meanwhile, genes related to lipolysis (*Atgl*, *Hsl*, and *Mgl*) and fatty acid oxidation (*Acox1*) showed a significant increase in their levels in MC-supplemented animals compared with obese + vehicle animals. No changes were observed in *Cpt1b* levels or the glucose transporter *Glut1*, whereas a strong tendency towards a reduced expression of *Glut4* was observed in the obese + MC mice. 

To corroborate whether MC supplementation increases non-shivering thermogenesis, infrared thermography was applied to analyze the temperature of the animal’s back surface covering the interscapular BAT (Figure 4F,G), indicating an increased temperature after MC supplementation compared with the obese + vehicle group.

## 3. Discussion

The prevalence of obesity has increased due to westernized diets and sedentary lifestyles, and it is strongly related to other metabolic disorders [27]. It is well-established that an improvement of adipose tissue function could be a therapeutic target to trigger an amelioration in the development of these diseases [3,7,28,29]. However, there is a lack of therapeutic solutions to combat obesity. Recently, we described the combination of NAC, NR, LC, and betaine as a nutraceutical treatment that ameliorates NAFLD development [30]. However, the effect of this specific combination on other diseases has not been described. It has been shown how these individual metabolic cofactors could have an important effect on adipose tissue in obesity. Betaine has been reported to activate Akt, enhancing insulin sensitivity in adipose tissue [12]. Moreover, betaine can increase lipid oxidation and mitochondrial function in adipose tissue [15]. L-carnitine is essential to the correct transport of FFAs into the mitochondria for β-oxidation [14]. N-acetylcysteine has been linked to increased expression of thermogenic genes and improved glucose and insulin tolerance [10], and NR to improved insulin sensitivity through the regulation of sirtuin activity [11]. The present study investigated how the combination of these four metabolic cofactors could help to improve adipose tissue dysfunction, glucose tolerance, and insulin sensitivity in a diet-induced obese mouse model. It was found that MC-supplemented mice showed a reduction in body weight gain compared to the obese group. This result agrees with other studies in which betaine or L-carnitine supplementation decreased body weight [15,31,32]. A supplementation combining L-carnitine and NR also reduced body weight [21], and NAC also reduced body weight in different studies using mice with diet-induced obesity [33,34]. Interestingly, total and LDL cholesterol levels were reduced in MC-supplemented mice, confirming the effect of MC on lipid metabolism. These results of cholesterol levels correlate with the results found in different preclinical studies involving the ingredients used in the MC treatment [31,34]. 

The reduction of body weight due to MC supplementation was linked to amelioration in visceral adiposity, mimicking previous results in other preclinical studies [22,35,36]. Increased VAT can secrete adipokines and cytokines, leading to a pro-inflammatory state and causing IR and metabolic syndrome. Hence, a reduction of VAT can provide a significant benefit for the metabolism [37]. Indeed, indirect calorimetry confirmed that MC supplementation can increase oxidative metabolism and fat oxidation. These results correlate with the increased oxidative capacity observed in an obese model after betaine or NR supplementation [19,38]. Moreover, L-carnitine increased lipid oxidation in a clinical trial [39]. Thus, MC influences energy homeostasis and accounts for the reduction of adiposity in VAT depots. In contrast, the measurement of energy expenditure did not show the differences observed in RQ. These results are in concordance with other similar studies [40,41,42], which may indicate that the observed increase in fat oxidation after MC supplementation is probably more related to peroxisomal β-oxidation than to mitochondrial oxidation [43,44,45].

MC supplementation reduces the increase of visceral fat pads in obese mice by diminishing adipocyte size, with a decrease in the largest adipocytes and a tendency for the smallest adipocytes to increase, which correlates with the effect observed with L-carnitine supplementation in obese mice [46]. These results suggested an amelioration in adiposity, attributable to an increase in lipid oxidation. Similar results in terms of VAT were also reported using these ingredients separately in preclinical obese models [31,33,34]. In the case of NR, an NR precursor showed a reducing effect on adipocyte area in EWAT [47].

To look deeper into the molecular mechanisms regulated by MC supplementation in adipose tissue amelioration, genes related to main adipose functions were analyzed [5]. Adipose lipolysis occurs after hydrolysis of triglycerides to FFA and glycerol by the consecutive action of Atgl, Hsl, and Mgl; with the contribution of Plin1 in the Atgl activation [48]. Thus, MC supplementation showed a boosting effect on *Atgl* and *Plin1* expression, which may indicate an amelioration of adipose tissue function. Accordingly, individual betaine or L-carnitine treatments up-regulated *Atgl* expression in adipose tissue [49]. In addition, this increase in lipolysis could be related to the increment of fatty oxidation due to MC supplementation, as observed in the *Acox1* expression results that correlate with those results obtained in the indirect calorimetry and for the reduction of fat mass. Moreover, similar effects were also observed in a diet-induced obese study with betaine supplementation [50]. 

Moreover, the reduction of BAT weight observed with MC supplementation was consistent with other studies in which the ingredients of MC were used separately [10,51,52]. In addition, BAT weight reduction through MC supplementation was linked to a reduction in the number and size of lipid droplets in BAT after MC supplementation. This reduction of large lipid droplets correlates with preceding studies of NAC or L-carnitine supplementation [10,51]. Moreover, mRNA expression analysis showed that genes related to lipolysis and fatty acid oxidation were also increased by MC supplementation, which can explain the increased oxidation of lipids in BAT and is coherent with previous studies using the ingredients of MC supplementation that managed to increase the expression of these genes in the adipose tissue, as described above [48,49,50]. Furthermore, increased thermogenesis by MC supplementation was verified through the significantly raised expression of certain thermogenic BAT genes, consistent with the capabilities of NR, L-carnitine, and NAC [34,51,52]. These results can be linked with increased BAT thermogenesis in MC-supplemented mice, which was previously reported in previous studies using one of the ingredients in the MC [10,51,52], and they confirm the improvement of BAT activity through MC supplementation.

IR is an important risk factor in the development of adipose tissue in obesity [53]. Fasting levels of glucose and insulin, HOMA-IR, GTT and ITT showed significant amelioration after MC supplementation. These results correlate with preclinical studies using HFD mice models treated separately with betaine, NAC, and NR, in which glucose and insulin tolerance were improved [10,50,54]. To understand the increased insulin sensitivity, gene expression analysis was performed for the main adipose glucose transporters [55,56]. *Glut1* expression levels did not show any significant difference between groups in EWAT or BAT. In contrast, *Glut4,* which is regulated by insulin, was up-regulated in the EWAT of the MC-supplemented group, but not in the BAT, which could indicate an increase in glucose uptake into visceral adipose tissue. That correlates with a previous study of L-carnitine supplementation in rats with a metabolic syndrome, where *Glut4* was up-regulated, and insulin sensitivity was improved [55]. Moreover, betaine was found to improve *Glut4* expression through increasing AMPK activation [57], and a precursor of NR improved adipose gene expression of *Glut4* through the same mechanism [58].

There were some limitations in this study. One of them was its design as a 4-week MC treatment on obese mice, as a corrective evaluation against obesity. However, a long-term study would have been of value to elucidate where obesity and lipid expression patterns can be reversed or modified more effectively. In addition, the possibility of shorter times or other doses should be evaluated to determine whether the effect is immediate or needs more time to be effective. However, we chose this study design because using this 4-week “corrective” evaluation we have seen good results in both mice and hamster studies [30,59,60]. Given the promising results obtained, it would be very interesting to carry out further studies to assess the potential of this combination of metabolic cofactors as a preventive treatment for obesity. Another potential limitation of the present study is that MC supplementation was applied only to obese animals. Therefore, further studies are needed to discern the effects of MC treatment on other metabolic disturbances, as well as to compare the impact of MC supplementation on obesity in lean animals.

To sum up, MC supplementation promotes the amelioration of adipose tissue dysfunction observed in the diet-induced obese mouse model. This study demonstrated the implication of the metabolic cofactors in the different pathways affected in the adipose tissue during metabolic dysfunction, involving a reduction of body weight and visceral adiposity, decreasing fat depots, improved BAT activity, and improved circulatory biomarkers of obesity, such as total cholesterol and LDL-cholesterol. Furthermore, the MC promoted lipid oxidation through the upregulation of genes related to lipolysis and fatty acid oxidation, in addition to the increased thermogenic gene expression in BAT. Finally, MC supplementation displayed beneficial effects on insulin sensitivity, improving blood insulin and glucose levels and up-regulating *Glut4*. Altogether, this study suggests that MC supplementation ameliorates adipose tissue dysfunction acting in different pathways affected by obesity, and can be an effective treatment for reducing the incidence of obesity-related metabolic disorders. 

## 4. Materials and Methods

### 4.1. Animal Model

Groups of 6-week-old male C57BL/6J mice (Envigo, Sant Feliu de Codines, Spain) were housed under controlled conditions of temperature (22 ± 2 °C) and humidity (55 ± 10%) and a 12-h light/dark cycle, with free access to food and water. Animals were fed with a high-fat diet (D12331, Research Diets, New Brunswick, NJ, USA) supplemented with 23.1 g/L fructose and 18.9 g/L sucrose in the drinking water. Mice were kept on these diets for 20 weeks in ad libitum conditions. All experimental protocols were approved by the Animal Ethics Committee of the Technological Unit of Nutrition and Health of Eurecat (Reus, Spain), and the Generalitat de Catalunya approved all the procedures (10281). The experimental protocol followed the “Principles of Laboratory Care” guidelines and was carried out following the European Communities Council Directive (2010/63/EEC). From the 16th to 20th week, obese mice were randomly distributed into two groups: 8 mice were kept under the same feed conditions described before (obese + vehicle group), and 8 mice were supplemented with a combination of metabolic cofactors (obese + MC group). The MC comprised a mix of the following compounds: 400 mg/kg of LC tartrate (Cambridge Commodities, Ely, UK), 400 mg/kg NAC (Cambridge Commodities, Ely, UK), 800 mg/kg betaine (Cambridge Commodities, Ely, UK), and 400 mg/kg NR (ChromaDex, Los Angeles, CA, USA). LC was administrated through LC tartrate (LCT) containing 68.2% LC, providing 560 mg/kg to reach the dose of 400 mg LC/kg. Betaine, LCT, NAC, and NR were diluted with drinking water. These specific doses were determined based on previous studies and a calculation of dose translation from human to animal [61]. Solutions were freshly prepared three times per week from stock powders and protected from light. Bodyweight and food intake data were recorded once a week during the entire study. In week 19, insulin and glucose tolerance tests (ITT and GTT respectively) were performed (see below). In the 20th week, animals were sacrificed, being deprived of food for 8 h before being euthanized. Blood was collected and serum was obtained by centrifugation and stored at −80 °C for further analysis. Brown adipose tissue (BAT) and white adipose tissue (WAT) depots (inguinal (IWAT), epididymal (EWAT), retroperitoneal (RWAT), and mesenteric (MWAT)) were immediately collected, weighed, and snap-frozen in liquid nitrogen to be kept at −80 °C for further determinations or fixed to perform histological analyses.

### 4.2. Serum and Blood Analysis

Serum fasting glucose, total cholesterol, and triglycerides (QCA, Barcelona, Spain) were analyzed by enzymatic colorimetric assays after sacrifice. Serum fasting insulinemia was analyzed using an insulin ELISA kit (Mercodia, Uppsala, Sweden), and serum HDL and LDL levels were analyzed using an EnzyChrom ™ AF HDL and LDL/VLDL assay kit (BioAssay System, Hayward, CA, USA). Lastly, LDL/HDL ratio was calculated. HOMA-IR (homeostatic model assessment for insulin resistance), which is an assessment that estimates insulin resistance in terms of beta-cell function and insulin sensitivity, was calculated from circulating levels of fasting glucose and insulin (glucose (mmol/L) × insulin (UI/L) × 22.5) [25].

For the GTT, during the third week of supplementation, mice fasted overnight and after fasted blood glucose levels, were measured, mice were injected i.p. with 1.5 g glucose/kg body weight (Merck KGaA, Darmstadt, Germany), and blood glucose levels were measured every 30, 60, 90, 120, 150, and 180 min, collecting blood from the mice’s tails. For the ITT, during the fourth week of treatment, mice fasted for 6 h and baseline levels of blood glucose were measured using a standard glucometer (LifeScan, Milpitas, CA, USA). Animals were then injected (i.p. 0.375 mU/g of body weight) with human rapid insulin (Actrapid^®^ Innolet^®^, Novo Nordisk A/S, Bagsvaerd, Denmark), and blood glucose levels were measured as explained in the GTT test process above.

### 4.3. Indirect Calorimetry

Indirect calorimetry analyses were performed 2 weeks before all mice were sacrificed. An OxyletProTM system (PANLAB, Cornellà, Spain) was employed to perform calorimetry. Mice were left in acrylic boxes with free access to their diet and vehicle or treatment. After an acclimation period of 3 h, oxygen consumption (VO_2_) and carbon dioxide production (VCO_2_) were measured every 9 min by an O_2_ and CO_2_ analyzer at a constant flow rate of 600 mL/min. The respiratory quotient (RQ) as the VCO_2_/VO_2_ ratio was calculated by Metabolism 2.1.02 software (PANLAB, Cornellà, Spain). The fat rate was calculated using the VCO_2_ and the VO_2_ measures and applying the Frayn stoichiometric equations, which define fat oxidation rates as 1.67 × VO_2_ − 1.67 × VCO_2_ − 1.92 n (g/min) [62]. A nitrogen excretion rate (n) of 135 µg kg^−1^ min^−1^ was assumed. Finally, fat oxidation level was obtained using the Atwater general conversion factor. The fat rate was multiplied by 37 [63]. Total energy expenditure (EE), oxygen consumption (VO_2_), and carbon dioxide production (VCO_2_) were also calculated using the Metabolism 2.1.02 software (PANLAB, Cornellà, Spain). 

### 4.4. Histological Analysis 

EWAT and BAT portions fixed in buffered formalin (4% formaldehyde, 4 gr/L NaH_2_PO_4_, 6.5 gr/L Na_2_HPO_4_; pH 6.8) were cut at a thickness of 5 µm and stained with hematoxylin & eosin (H & E). BAT and EWAT images (magnification 40×) were taken with a microscope (ECLIPSE Ti; Nikon, Tokyo, Japan) coupled to a digital sight camera (DS-Ri1, Nikon), and analyzed using ImageJ NDPI software (National Institutes of Health, Bethesda, MD, USA; https://imagej.nih.gov/ij, accessed on 25 July 2022, version 1.52a). To avoid any bias in the analysis, the study had a double-blind design, preventing the reviewers from viewing any data from the mice during the histopathological analysis. Area quantification of adipocytes was analyzed using the Adiposoft plugin to assess the state of EWAT between groups. Lipid droplet quantification in BAT was analyzed using the Droplet Finder plugin.

### 4.5. mRNA Extraction and Quantitative Polymerase Chain Reaction

According to the manufacturer’s instructions, homogenates from EWATs and BATs were used for total mRNA extractions with TriPure reagent (Roche Diagnostic, Sant Cugat del Vallès, Barcelona, Spain). mRNA concentration and purity were determined using a nanophotometer (Implen GmbH, München, Germany). RNA was converted to cDNA using the high-capacity RNA-to-cDNA Kit (Applied Biosystems, Wilmington, DE, USA). The cDNAs were diluted 1:10 before incubation with commercial LightCycler 480 Sybr green I master on a Lightcycler^®^ 480 II (Roche Diagnostics GmbH, Manheim, Germany). The relative gene expression levels were calculated using the 2^−ΔΔCt^ method [64]. Table 1 shows a list of primers used that were previously described in other studies and verified with Primer-Blast software (National Center for Biotechnology Information, Bethesda, MD, USA). *36b4* was used as a housekeeping gene [30].

### 4.6. BAT Temperature Measurements

The temperature surrounding the BAT was visualized using a high-resolution infrared camera (FLIR Systems) and analyzed with a dedicated software package (FLIR-Tools-Software, FLIR; Kent, UK), as previously described [79]. For each image, the area surrounding the BAT was delimited and the average temperature of the skin area was calculated as the average of 3 pictures for each animal.

### 4.7. Statistical Analysis 

Statistical analyses were performed using GraphPad Prism 9 software (Graph-Pad Software, La Jolla, CA, USA). Data are presented as mean ± SEM. Data distribution was analyzed by the Shapiro–Wilk normality test. Differences between the two groups were determined using an unpaired *t*-test (two-tailed, 95% confidence interval). A *p*-value below 0.05 was considered statistically significant.

## Figures and Tables

**Table 1:** Sequences of the oligonucleotides used in the RT-PCR.

Primers	Forward	Reverse	Reference
*Acc1*	GATGAACCATCTCCGTTGGC	CCCAATTATGAATCGGGAGTGC	[65]
*Acox1*	CTATGGGATCAGCCAGAAAG	AGTCAAAGGCATCCACCAAAG	[66]
*Atgl*	CAACGCCACTCACATCTACGG	GGACACCTCAATAATGTTGGCAC	[67]
*Cpt1* *α*	CTCAGTGGGAGCGACTCTTCA	GGCCTCTGTGGTACACGACAA	[68]
*Cpt1b*	CGAGGATTCTCTGGAACTGC	GGCCTCTGTGGTACACGACAA	[69]
*Dio2*	AGAGTGGAGGCGCATGCT	GGCATCTAGGAGGAAGCTGTTC	[70]
*Fasn*	GCTGCGGAAACTTCAGGAAAT	AGAGACGTGTCACTCCTGGACTT	[71]
*Glut1*	TCAACACGGCCTTCACTG	CACGATGCTCAGATAGGACATC	[72]
*Glut4*	AAAAGTGCCTGAAACCAGAG	TCACCTCCTGCTCTAAAAGG	[73]
*Hsl*	TCCTGGAACTAAGTGGACGCAAG	CAGACACACTCCTGCGCATAGAC	[74]
*Mgl*	CGGAACAAGTCGGAGGTTGA	TGTCCTGACTCCGGGATGAT	[67]
*Pgc1a*	AGCCGTGACCACTGACAACGAG	GCTGCATGGTTCTGAGTGCTAAG	[75]
*Plin1*	GTCAATGAACAAGGGCCCAAC	CACAGGCAGCTGCAGAACTCTC	[74]
*Ppar* *α*	CCCTGTTTGTGGCTGCTATAATTT	GGGAAGAGGAAGGTGTCATCTG	[76]
*Prdm16*	CAGCACGGTGAAGCCATTC	GCGTGCATCCGCTTGTG	[77]
*Scd1*	AGATCTCCAGTTCTTACACGACCAC	GACGGATGTCTTCTTCCAGGTG	[65]
*Ucp1*	ACTGCCACACCTCCAGTCATT	CTTTGCCTCACTCAGGATTGG	[78]
*36b4*	AGTCCCTGCCCTTTGTACACA	CGATCCGAGGGCCTCACTA	[30]

## Supplementary Materials

The following supporting information can be downloaded at: https://www.mdpi.com/article/10.3390/ijms232314923/s1.
