# Supplementary material for: Reduction of Obesity and Insulin Resistance through Dual Targeting of VAT and BAT by a Novel Combination of Metabolic Cofactors
Source: Int J Mol Sci. 2022 Nov 29;23(23):14923. doi: 10.3390/ijms232314923 (PMC9738317; doi:10.3390/ijms232314923)
Supplement: Supplementary file 1 [file ijms-23-14923-s001.zip › ijms-1955203-supplementary.pdf]

Supp Figure S1

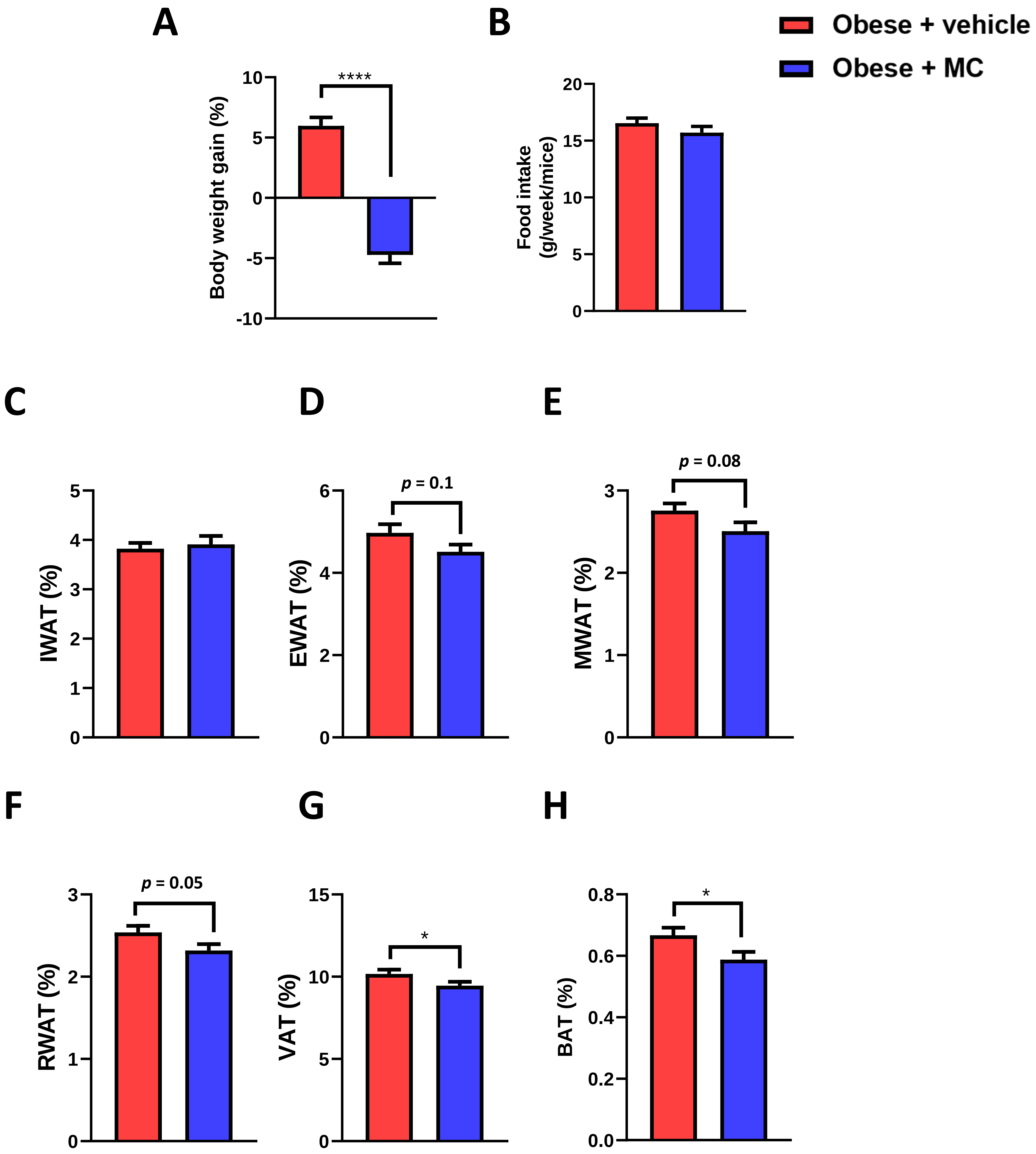

Figure S1. Obese mice supplemented with MC reduce body weight gain and presented smaller visceral WAT depots. Effects of MC treatment on: (A) percentage of body weight gain; (B) food intake, percentage from the total body weight of (C) IWAT (D) EWAT, (E) MWAT, (F) RWAT, (G) VAT and (H) BAT. Data are mean ± SEM. * $p < 0.05$, **** $p < 0.0001$.

Supp Figure S2

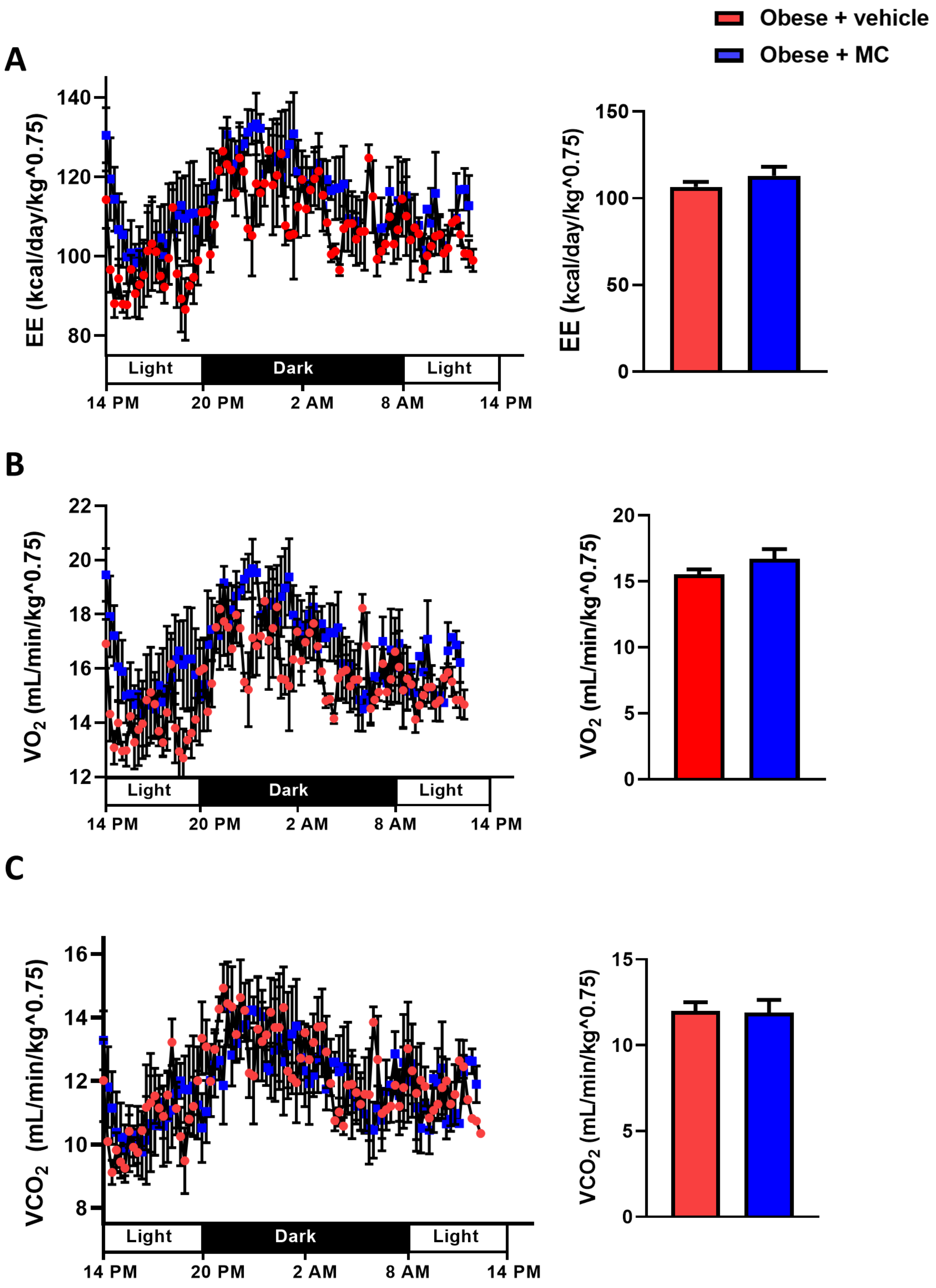

Figure S2. Effect of MC treatment on (A) energy expenditure (EE), (B) VO_2, and (C) VCO_2 during 24 h (left panels) and the mean of these measurement (right panels). Data are mean ± SEM.
